# Fractures

**Published:** 1901-01

**Authors:** 


Fractures.—By Carl Beck, M. D., Visiting Surgeon to St. Mark's Hospital and to the New York German Poliklinic, etc. With an appendix on the "Practical Use of the Roentgen Rays. 178 illustrations. Pp. 335. Price, $3.50. Philadelphia: W. B. Saunders & Co., Publishers, 1900.

This work treats the subject of fractures in the most modern way. The Roentgent ray has been employed in every fracture where its service is unquestioned, thereby making the work one of great practical value. The author has successfully combined a large experience in this department of medicine with an appreciation of all that is modern and useful and has mode that will take with the profession everywhere. T. J. B.

